# Patterns and predictors of workplace health promotion: cross-sectional findings from a company survey in Germany

**DOI:** 10.1186/s12889-015-1647-z

**Published:** 2015-04-09

**Authors:** David Beck, Uwe Lenhardt, Britta Schmitt, Sabine Sommer

**Affiliations:** Federal Institute for Occupational Safety and Health (BAuA), Nöldnerstraße 40-42, 10317 Berlin, Germany

**Keywords:** Workplace health promotion, Quality, Prevalence, Predictors, Company survey

## Abstract

**Background:**

Although the popularity of workplace health promotion (WHP) has considerably increased over the years, there are still concerns about the way this concept is being implemented by the companies. There is, however, a seeming lack of empirical knowledge about variations in WHP practice. Therefore, the aim of this study was to determine the prevalence of different patterns (and related quality levels) of WHP activity and the effect of organisational predictors on the chances of these WHP activity levels being implemented.

**Methods:**

Data from an establishment survey (N = 6,500) were used to calculate the prevalences of four configurations of WHP among German companies. Furthermore, multinominal logistic regressions were performed to determine odds ratios for these WHP activity levels according to several organisational characteristics.

**Results:**

9% of companies exhibited the most comprehensive type of WHP including analysis, individual-directed prevention measures and participatory groups concerned with working conditions improvement (level A), 18% featured a combination of analysis and individual-directed prevention (level B), 29% had reported measures from only one of these categories (level C), and 44% showed no WHP activity at all (level D). In the multivariate analysis company size turned out to be the strongest predictor of WHP at all levels. WHP was also predicted by a good economic situation of the company, the availability of safety specialist assistance, the availability of specialist assistance in occupational health and the presence of an employee representative body. These effects usually became stronger when moving up in the hierarchy of WHP levels. For the two sector-level variables (private vs. public, production vs. services) no statistically significant associations with WHP were found.

**Conclusions:**

WHP still shows great potential for improvement both in quantitative and qualitative terms. Especially required are additional efforts in developing and implementing WHP practice models and dissemination strategies which are tailored to the particular conditions and needs of small companies. However, findings suggest that the chances for achieving progress in WHP also depend on developments in adjacent policy areas such as labour relations or occupational safety and health.

## Background

In many countries, Workplace Health Promotion (WHP) has become a well-established concept in both the academic field and social policy [1]. This is also the case in Germany, where the statutory health insurance funds are legally bound to provide WHP services which cover activities such as analysing the companiesʼ health situation, developing proposals for its improvement and supporting the implementation of the suggested measures. The financial resources invested in WHP services have considerably increased over the years. In 2011, for instance, German health insurance funds spent roughly 42 million Euros on such services [2].

In theory, WHP is quite an ambitious concept. According to the Luxembourg Declaration of the European Network for Workplace Health Promotion [3], it is characterised not only by combining individual-directed and environment-directed measures but also by pursuing a resource-oriented approach (“enhancing health-promoting potentials and well-being in the workforce”) which expands the traditional concept of risk reduction. Moreover, WHP should be designed as a systematically managed continuous improvement process comprising needs analysis, priority setting, planning, implementation, continuous control and evaluation, and it should strongly emphasise worker participation in this process.

It has been argued that in practice, those criteria are met only to a rather limited extent, as WHP mostly occurs in the form of isolated measures directed to individual health behaviours [4-7]. However, empirical evidence for this point of view is fairly scarce. One of the few studies available in this context is based on an analysis of data from the 2004 U.S. National Worksite Health Promotion Survey [8]. It showed that only a small minority of companies had implemented a “comprehensive” type of WHP, incorporating all of the following elements: health education; supportive social and physical work environment; integration into the organisationʼs structure; linkage to related programmes; worksite health screening. Whether this finding is still valid for the U.S. or even other countries remains unclear. In recent years, several survey studies have examined the nationwide prevalence of various WHP measures or of WHP in general [9-14], but none of them differentiated between configurations of WHP measures indicating the comprehensiveness of WHP in practice. The study of Hollederer [9] merely refers to the sheer number of WHP measures accumulating at the company level, without any consideration of the actual types of measures being combined. There is one study from Germany featuring some kind of quality grading of WHP activity, but its findings are based on a rather small and selective sample of mid-sized to large companies from only two regions and branches [15].

Therefore, the aims of this study were (1) to identify combinations of WHP measures representing different development levels of WHP practice, (2) to estimate the overall prevalence of these WHP patterns across all German companies, and (3) to determine factors by which the chance of occurence of these WHP patterns is influenced.

## Methods

### Data source

The study consists in a secondary analysis of survey data collected from 6,500 companies with at least one employee in mid-2011 as part of the evaluation of the German Joint Occupational Safety and Health Strategy *(Gemeinsame Deutsche Arbeitsschutzstrategie – GDA)*. In the survey, a disproportionate stratified random sampling technique was used. In order to obtain a dataset which is representative with respect to company size, sector and region, disproportionalities were later readjusted by means of design weighting (weighting factors ranging between 0.01 and 14.274). The target persons (i.e., the highest-ranking company members with responsibilities in occupational safety and health coordination) responded to a questionnaire, administered by CATI, on a wide range of safety and health topics, including WHP. The response rate was rather low at 15% (which will be further discussed in the “Strengths and limitations” section of this article). The survey methodology has been described in more detail by TNS Infratest Sozialforschung [16].

### Dependent variables

The interviewees were asked about the availability of *six types of WHP measures* in their company (A: analysis of sick leave data, B: staff surveys on workplace health, C: in-house exercise programmes, D: health circles or similar participatory groups, E: addiction prevention programmes, F: individual health checks). Response categories were ‘yesʼ, ‘noʼ, ‘do not knowʼ and ‘not answered (n/a)ʼ. In order to simplify the set of variables, these six measures were attributed to three more general categories: (1) *analysis* (measures A and B), (2) *individual-oriented prevention* (measures C, E and F), (3) *health circles* (measure D). Health circles were given a separate category because they are designed to identify needs for workplace improvements in a participatory manner and may therefore indicate the presence of a more structure-oriented prevention approach within WHP [17].

Depending on whether - and how - these categories of WHP measures were combined, four levels of company WHP activity were determined which represent different degrees of approximation to a comprehensive WHP approach in practice:

- *Level A*: health circles combined with analysis or individual-oriented prevention, or both
- *Level B*: analysis and individual-oriented prevention combined, no health circles
- *Level C*: measures from only one category, no combinations
- *Level D*: no measures at all

### Independent variables

Several factors which have been previously demonstrated to affect company practice in prevention were covered in the GDA survey questionnaire and could therefore be included in the present study: company size [18], sector [19], economic situation [20], employee representation [21], and specialist occupational safety and health assistance [22].

C*ompany size* was determined by the question “How many employees, approximately, are working in your company?” The information obtained was categorised as follows: ‘1–9 employeesʼ, ‘10–49 employeesʼ, ‘50–249 employeesʼ, ‘ ≥ 250 employeesʼ.

*Sector* was measured in two ways: first by the question “Does your establishment belong to the public service sector?” (‘yesʼ; ‘no, private businessʼ; ‘do not knowʼ; ‘n/aʼ), and second by using a dichotomous categorisation of the companies’ branch affiliations (‘production/agricultureʼ, ‘servicesʼ).

The *economic situation* of the surveyed organisations was measured by one question: “How do you rate the current economic situation (public service: “budgetary situation“) of your company (public service: “of your establishment”)?” (‘goodʼ, ‘satisfactoryʼ, ‘badʼ, ‘do not knowʼ, ‘n/aʼ).

Respondents were also asked about the presence of an *employee representative body* (“works council”) in their company (‘yesʼ, ‘noʼ, ‘do not knowʼ, ‘n/aʼ). If data analyses related to this variable were carried out, ‘5–9 employeesʼ was used as the lowest size category, as legal regulations on works councils in Germany do not apply to companies smaller than that.

Companies should further indicate if they make use, as required by law, of *safety specialist assistance* (‘yesʼ, ‘noʼ, ‘do not knowʼ, ‘n/aʼ). Small companies (up to 50 employees) were asked if they have opted for an alternative model in which company owners may themselves perform the tasks of professional occupational safety and health specialists after finishing special trainig courses (‘yesʼ, ‘noʼ, ‘do not knowʼ, ‘n/aʼ). If this was the case (or if the respondent himself was a safety engineer), the company was automatically classified as employing safety specialist assistance.

*Specialist assistance in occupational health* was measured similarly (“Do you make use of an occupational physician’s assistance when carrying out your duties in occupational safety and health?” [‘yesʼ, ‘noʼ, ‘do not knowʼ, ‘n/aʼ], or: “Do you participate in the alternative assistance model?” [‘yesʼ, ‘noʼ, ‘do not knowʼ, ‘n/aʼ]).

### Statistical analyses

Descriptive analyses of weighted data were carried out by using the CSTABULATE procedure from the SPSS statistical software package 18.0 for Windows. Multinominal logistic regressions based on unweighted data were performed to determine odds ratios (OR) for different levels of WHP activity according to company size, sector, economic situation, employee representation and specialist occupational safety and health assistance. In this context, an OR indicates the chance that a subgroup of companies exhibits a given WHP level rather than showing no WHP activity at all, in relation to the chance found in the reference group. For the multivariate analyses, the NOMREG procedure from SPSS 18.0 was used.

## Results

The frequencies of the studied variables (absolute unweighted numbers and weighted percentages) are shown in Table 1. Just like the basic population, the study sample largely consists of small companies with up to 50 employees. Together, mid-sized and large companies account for not more than 6%. The vast majority – well over 90% – of the responding organisations belong to the private sector, three out of four are located in services. The companies’ economic situation was predominantly rated as either good or, to a somewhat lesser extent, satisfactory, only 7% of respondents regarded it as bad. Just about one out of six companies has an employee representative body, specialist assistance in safety and in occupational health is available in 59% and 48% of cases, respectively.

**Table 1:** **Descriptive statistics for the variables included**

**Variable**	**n (unw)**	**% (w) (95% CI)**
**Number of employees**		
1–9	1,815	71 (69–73)
10–49	1,878	24 (22–26)
50–249	1,715	5 (4–5)
≥250	1,092	1 (1–1)
**Sector (I)** ^**a**^		
Private	5,456	92 (90–93)
Public	1,031	8 (7–10)
**Sector (II)**		
Services	4,279	76 (74–78)
Production/agriculture	2,221	24 (22–26)
**Economic situation** ^**a**^		
Bad	554	7 (6–8)
Satisfactory	2,210	43 (40–45)
Good	3,522	50 (48–53)
**Works council** ^**b**^		
Not yes	3,019	84 (82–86)
Yes	2,447	16 (14–18)
**Safety specialist assistance**		
Not yes	1,311	41 (39–44)
Yes	5,189	59 (56–61)
**Occupational health specialist assistance**		
Not yes	1,800	52 (49–54)
Yes	4,700	48 (46–51)
**Workplace health promotion measures**		
Analyses of sick leave data (yes)	2,967	22 (20–24)
Staff surveys on workplace health (yes)	2,552	28 (25–30)
In-house exercise programmes (yes)	1,438	12 (10–13)
Addiction prevention programmes (yes)	1,512	9 (8–11)
Individual health checks (yes)	3,004	31 (29–33)
Health circles or similar groups (yes)	1,327	11 (10–13)
**Total**	6,500	100

n = number of responses; unw = unweighted; w = weighted; CI = confidence interval.

^a^companies falling within response categories ʻdo not knowʼ and ʻn/aʼ not included, hence N < 6,500; ^b^only companies with ≥ 5 employees (N = 5,466).

Among the WHP measures studied, individual health checks and staff surveys on workplace health, although individually present in less than one-third of the companies, are the most prevalent, followed by analysis of sick-leave data (22%), in-house exercise programmes (12%), health circles and similar participatory groups (11%), and addiction prevention programmes (9%).

### Prevalences of different configurations of WHP measures

Table 2 displays the unweighted number of cases and the weighted percentages for each of the four WHP configurations described above. 44% of the companies did not report any of the WHP measures they were asked about (level D). 29% had implemented measures from only one category (level C), either analysis (13%) or individual-oriented prevention (14%) or health circles (2%). In 18% of the companies, analysis was combined with individual-oriented prevention (level B). A combination of health circles with analysis and/or individual-oriented prevention (level A) was found in less than one out of ten companies.

**Table 2:** **Prevalences of four different WHP configurations**

**WHP configuration**	**n (unw)**	**% (w) (95% CI)**
Level D (no measures)	1,644	44 (42–47)
Level C (measures from only one category)	1,808	29 (27–31)
*Specifically:*		
Analysis only	930	13 (12–15)
Individual-oriented prevention only	824	14 (12–15)
Health circles only	54	2 (1–3)
Level B (analysis and individual-oriented prevention combined)	1,775	18 (16–20)
Level A (health circles and analysis and/or individual-oriented prevention combined)	1,273	9 (8–11)
**Total**	6,500	100

N = number of responses; unw = unweighted; w = weighted; CI = confidence interval.

As can be seen from Table 3, the prevalences of the four WHP levels differ considerably between subgroups of the sample. The “no measures” level (D) is particularly common (51%) in micro-companies (1–9 employees) and, to a lesser extent, in small companies with 10–49 employees. These, in turn, feature the highest prevalence of WHP level C (amounting to a good third of the companies), followed by mid-sized companies (50–249 employees) (29%), micro-companies (27%) and, clearly lagging behind, large companies (13%). Mid-sized and large establishments are the most active ones at level B and, even more pronounced, level A, which occurs in just over half of the companies with 250 and more employees but only in about one out of ten small companies.

**Table 3:** **Prevalences of WHP configurations, by subgroups of the sample**

**Variable**	**Level D**		**Level C**		**Level B**		**Level A**	
	**% (w)**	**95% CI**	**% (w)**	**95% CI**	**% (w)**	**95% CI**	**% (w)**	**95% CI**
**Number of employees**								
1–9	51	(48–55)	27	(24–30)	14	(12–17)	8	(6–10)
10–49	30	(27–34)	36	(32–39)	25	(22–28)	10	(8–12)
50–249	11	(9–14)	29	(26–32)	37	(33–40)	23	(20–26)
≥250	3	(2–4)	13	(10–15)	32	(28–36)	53	(49–57)
**Sector (I)**								
Private	46	(44–49)	29	(26–31)	17	(15–19)	8	(7–9)
Public	22	(26–29)	30	(24–38)	25	(19–32)	23	(17–31)
**Sector (II)**								
Services	45	(42–48)	27	(24–29)	18	(16–20)	10	(9–12)
Production/agriculture	41	(36–45)	35	(31–40)	18	(15–22)	6	(5–8)
**Economic situation**								
Bad	52	(43–61)	25	(18–32)	15	(10–23)	8	(4–16)
Satisfactory	47	(43–51)	29	(25–33)	16	(14–20)	8	(6–10)
Good	41	(38–45)	29	(26–32)	20	(17–22)	11	(9–13)
**Works council** ^a^								
Not yes	42	(39–46)	31	(28–34)	20	(17–22)	7	(6–9)
Yes	11	(8–15)	29	(24–34)	37	(31–42)	24	(20–28)
**Safety specialist assistance**								
Not yes	60	(56–64)	26	(23–30)	9	(7–11)	5	(4–7)
Yes	33	(30–36)	31	(28–33)	24	(22–27)	12	(10–14)
**Occupational health specialist assistance**								
Not yes	58	(54–62)	27	(24–31)	10	(8–12)	5	(4–7)
Yes	30	(26–33)	30	(27–33)	27	(24–30)	14	(12–16)

N = 6,500; w = weighted; CI = confidence interval; ^a^only companies with ≥5 employees (N = 5,466).

WHP, especially the highest level, is clearly more prevalent in the public than in the private sector, where just about 8% (public: 23%) of companies reported activities conforming to level A. Differences between the production and the services sector are much smaller in comparison, the former being slightly more active in WHP, if only at level C. WHP prevalence is also higher in companies rating their economic situation as ‘goodʼ than in those with less favourable appraisals. Level C aside, this is also true for companies where works councils are present (as opposed to those without employee representation). Finally, organisations making use of specialist assistance in safety and/or in occupational health exhibit more WHP, at any level, than organisations which do not.

### Predictors of WHP activity at different levels

According to the results of our multivariate analyses (shown in Table 4), company size predicts WHP activity at all levels, the chance of activity being higher the bigger the companies are. Odds ratios for large companies (≥250 employees, reference: 5–9 employees) were 3.7 (level C), 10.1 (level B) and 21.5 (level A). Good economic situation, presence of a works council, availability of safety specialist assistance and availability of specialist assistance in occupational health were each independently associated with WHP activity at all levels as well, although not as strongly as company size. In contrast, none of the sector variables showed any effect on WHP activity in the multivariate model, irrespective of level. Accordingly, the goodness of fit of the model does not significantly change when the sector variables are being eliminated (Nagelkerkeʼs pseudo-*R*^*2*^: 0.321 for the original, 0.319 for the reduced model).

**Table 4:** **Results of the multinomial logistic regression analysis**

**Variable**	**WHP level C**			**WHP level B**			**WHP level A**		
	**OR**	**95% CI**	***p***	**OR**	**95% CI**	***p***	**OR**	**95% CI**	***p***
**Number of employees**									
5–9 (Ref.)	1.00			1.00			1.00		
10–49	1.50	1.22–1.86	<0.001	2.00	1.55–2.59	<0.001	1.16	0.83–1.62	0.386
50–249	2.78	2.12–3.66	<0.001	4.55	3.37–6.15	<0.001	3.75	2.60–5.40	*˂*0.001
≥250	3.72	2.25–6.14	<0.001	10.14	6.15–16.73	<0.001	21.52	12.60–36.72	*˂*0.001
**Sector (I)**									
Private (Ref.)	1.00			1.00			1.00		
Public	1.21	0.91–1.62	0.195	1.00	0.75–1.35	0.994	1.09	0.79–1.49	0.602
**Sector (II)**									
Services (Ref.)	1.00			1.00			1.00		
Production/agriculture	1.18	0.98–1.42	0.075	0.99	0.81–1.20	0.891	0.91	0.73–1.14	0.401
**Economic situation**									
Bad (Ref.)	1.00			1.00			1.00		
Satisfactory	1.10	0.80–1.51	0.541	1.38	0.98–1.94	0.067	1.82	1.24–2.67	0.002
Good	1.37	1.00–1.88	0.048	2.26	1.62–3.17	<0.001	2.84	1.95–4.15	*˂*0.001
**Works council**									
Not yes (Ref.)	1.00			1.00			1.00		
Yes	1.62	1.26–2.09	<0.001	2.20	1.71–2.82	<0.001	4.07	3.09–5.37	*˂*0.001
**Safety specialist assistance**									
Not yes (Ref.)	1.00			1.00			1.00		
Yes	1.55	1.23–1.97	<0.001	2.50	1.85–3.38	<0.001	1.62	1.12–2.35	0.011
**Occupational health specialist assistance**									
Not yes (Ref.)	1.00			1.00			1.00		
Yes	1.45	1.18–1.79	0.001	2.92	2.28–3.73	<0.001	3.08	2.22–4.27	*˂*0.001

N = 5,267 companies with more than four employees; Nagelkerkeʼs pseudo-*R* ^*2*^ = 0.321; OR = odds ratio, indicates the chance that a subgroup of companies exhibits a given WHP level rather than showing no WHP activity at all (= WHP level D), in relation to the chance found in the reference group; CI = confidence interval.

For each of the variables associated with WHP (except safety specialist assistance, the effect of which is strongest at level B) a steady increase of ORs can be observed when proceeding from WHP level C to level A. At the same time, effect sizes increasingly diverge between the predictor variables: ORs range from 1.4 to 3.7 for WHP level C, from 2.2 to 10.1 for level B, and from 1.6 to 21.5 for level A. Moreover, the rank order of predictors according to their effect sizes varies from level to level: Company size (which is the most important predictor at all WHP levels) left aside, safety specialist assistance and works councils have the strongest effect on WHP level C, closely followed by occupational health specialist assistance and economic situation. The chance of activity level B is influenced most strongly by occupational health specialist assistance, followed by (in descending order) safety specialist assistance, economic situation and works councils. The latter, in turn, represents the strongest predictor of WHP level A, exhibiting a higher OR than occupational health specialist assistance, economic situation and safety specialist assistance, which has the weakest effect on WHP of this type.

## Discussion

To our knowledge this is the first study to examine the prevalence of different configurations of WHP measures as well as the significance of organisational factors in predicting corresponding levels of WHP comprehensiveness on a representative empirical basis. Results show that WHP has become fairly common among German companies, with the majority exhibiting at least some kind of measure in this field. The overall WHP prevalence rate of 56% may be lower than the figures reported from the U.S. (90% [23]) or from Denmark (82% [24]), but is still quite remarkable.

However, findings also suggest that these activities mostly fall short of what has been described as the essential characteristics of WHP, such as systematic planning and management, a participatory approach, or integration of individual and work environment oriented measures. Of all companies reporting WHP, 52% confine their activities to only one of the three categories of measures considered in this study. 29% abstain from analysing or collecting data which could help establish the needs for preventive action whereas 23% do only this, without taking any real preventive measures. Not more than two out of ten WHP-active companies show that they deal with working conditions and employee participation in the context of WHP, as indicated by reporting health circles (mostly in combination with analysis and/or individual-directed prevention measures). Thus, the results of our study corroborate the notion that a comprehensive type of WHP is still rather the exception than the rule.

Furthermore, our findings are consistent with evidence from previous studies [25-27] showing that the chances for WHP implementation – especially as far as more elaborate forms of WHP activity are concerned – are significantly reduced in small and economically threatened companies. The latter is easily understood by the fact that in situations of businesss hardship budgetary restrictions are sharp and the management’s attention is largely focussed on how to restabilise the market position of the company in the short term, leaving very little scope for activities (such as WHP) which are unlikely to pay off immediately. As to small establishments, the conditions for WHP implementation are not generally unfavourable since organisational structures are easier to overview, internal administrative barriers are few and social interaction is rather close, which clearly facilitates communication and mutual motivation. These advantages, however, seem to be mostly outweighed by a number of counteracting factors such as shortage of financial and personnel resources, lack of expertise, often high staff turnover and short company life span, or limited access to WHP service providers. Concentration of most, if not all, managerial responsibilities in a single person – i.e., the company owner –, which is a typical feature of many small firms, may also lead rather quickly to task overload and, consequently, to a higher probability of neglecting supposedly “soft issues” like WHP [28,29].

Previous studies found public services to be much more active in WHP than the private sector of the economy [25,30]. These findings are confirmed by our bivariate results according to which public establishments show a 44% higher general WHP prevalence rate and an almost threefold prevalence rate of WHP level A. The difference might be largely explainable by the fact that average company size is considerably greater [31], employee representation bodies are more frequent [32] and availability of specialist occupational safety and health assistance is higher in public services (authorsʼ calculations, data not shown), with each of these factors increasing the chance of WHP activity. If this is taken into account by performing multinominal logistic regression analysis, an association between public sector affiliation and WHP activity is no longer detectable.

Finally, the results of this study indicate that the presence of employee representation structures and the availability of professional safety and health expertise at the company level play an important role in facilitating the implementation of WHP measures, independently of other factors such as establishment size, sector, or economic situation of the company. Associations between the presence of a works council and WHP activity have been already demonstrated in previous studies, although it must be pointed out that these were limited either by the lack of multivariate analyses [9] or by focussing on a single branch [33]. Our finding that employing occupational physiciansʼ assistance increases the chance of WHP measures is contradictory to the latter study, which was, however, not only narrower in scope but also looking at a slightly different kind of outcome (“workplace health management”). Regarding safety specialists, the findings of our research are in fact novel as no other study to date seems to have investigated the significance of this group of experts in predicting WHP activity.

### Strengths and limitations

Concerning strengths it can be pointed out that the study is based on data from a comparatively large sample of companies which is not only representative for all of German companies with at least one employee in regard to establishment size, branch and region but also allows for rather differentiated analyses with statistically meaningful results. The validity of the findings clearly benefits from the fact that data were obtained from company managers or functionaries, as these are, due to their decision-making and coordinating responsibilities, likely to be better informed about the organisationsʼ preventive activities than ordinary employees. Furthermore, the study stands out from most of the other survey-based research on WHP for determining configurations of measures which are indicative of WHP comprehensiveness, rather than merely analysing distributional patterns of different individual measures.

Still, several limitations should be mentioned. It is a well-known fact that non-enforced business surveys tend to feature rather low response rates [34]. This problem is particularly pronounced in the survey our study is based on. One possible reason is that the particular subject of this survey – i.e., “safety and health at the workplace” – normally might not attract as much of a companyʼs attention as other subjects which are more closely business-related (e.g., market developments, technological innovations, tax policy issues). Moreover, companies in Germany seem to be less willing to participate in such a survey than companies in most other European countries, as figures from the European Survey of Enterprises on New and Emerging Risks (ESENER) show: in this survey, only 5 out of 31 countries had a lower response rate than Germany, where it was 18% [35].

The low response rate of the GDA-survey, whatever the reasons may be, certainly brings up the question of non-response bias. Bias effects related to the over-representation of – mostly less WHP-active – small and non-public establishments among non-responders have been compensated for by non-response adjustment of sample weights. Nevertheless, significant residual bias cannot be ruled out as it may be assumed that companies lacking awareness and activity in the field of occupational health (including WHP) were generally more likely to refuse participation in the survey. Therefore, a tendency towards overestimating the prevalence of WHP, especially of its higher levels, must be taken into account. While the reported WHP prevalences as such must be treated with caution for methodological reasons, it is unlikely, however, that our findings concerning the relative importance of different WHP comprehensiveness levels and the associations between these and the predictor variables are substantially biased by the low response rate of the survey. It goes without saying that the cross-sectional design of our study prohibits drawing any causal inferences from the associations found.

Finally, it must be noted that WHP practice was only partially captured by the survey questionnaire, which addressed a broad range of occupational health and safety issues, leaving room for not more than six sub-items related to WHP. Some established elements of WHP programmes, such as measures to promote healthy diet or stress management training, were not included, and no information on organisational and procedural modalities of WHP (e.g., integration into the company’s organisation; planning and management; responsibilities; resources; duration, intensity and scope of measures) was collected. Accordingly, this study provides only a rough approximation of WHP practice and its comprehensiveness. The same is true for predictors of WHP, as several organisational features which might be relevant in this regard – such as management approaches [36,37] or social capital [38] – were not within the scope of this study. To gain deeper insight into WHP activity, a more detailed empirical assessment of its structures and determinants, preferably through representative surveys clearly focussing on the particular subject of WHP, is strongly required in future research.

## Conclusions

From the results of this study it is evident that WHP, despite its increased popularity among companies, still shows great potential for development both in quantitative and qualitative terms. Most challenging in this regard is undoubtedly the small company sector where WHP is clearly less prevalent and comprehensive. Given that in Germany, as similar to the other EU countries, small organisations make up well over 95% of all companies (covering roughly 40% of the total workforce) [39], raising the low WHP activity level in this sector of the economy should be a high priority for national health promotion policies. This requires additional efforts in developing and implementing WHP practice models and dissemination strategies which are specially tailored to the particular conditions and needs of small companies. It should be kept in mind, however, that the chances for achieving progress in WHP also depend on developments in adjacent policy areas such as labour relations or occupational safety and health. As our findings suggest, reinforcement of worker representation structures at company level and strengthening professional OSH expert utilisation – even if not directly connected to, or driven by, WHP concerns – would be clearly beneficial for WHP.

### Ethical approval and consent

The study has not been submitted to an ethics committee because the internal ethical guidelines of the Federal Institute for Occupational Safety and Health do not require this for secondary analyses of survey data.

The participants were informed about the purpose of the survey and participation was voluntary (for details see [16]).

### Standards of reporting

The study was reported according to the STROBE guideline for reporting observational studies. For details see WEBLINK: http://www.equator-network.org/reporting-guidelines/strobe/.

### Data availability

Data available from GESIS Leibniz Institute for the Social Science, Data Catalogue DBK, Study No. ZA5634, DOI:10.4232/1.11483, Weblink: https://dbk.gesis.org/dbksearch/sdesc2.asp?no=5634&search=GDA%20Dachevaluation&search2=&DB=d&tab=0&notabs=&nf=1&af=&ll=10.

Data and documents are only released for academic research and teaching after the data depositor’s written authorisation. For this purpose the Data Archive obtains a written permission with specification of the user and the analysis intention.

## Abbreviations

CATI: Computer-assisted telephone interview
EU: European Union
GDA: Gemeinsame Deutsche Arbeitsschutzstrategie
OR: Odds ratio
OSH: Occupational safety and health
U.S: United States
WHP: Workplace health promotion
